# Hand hygiene with hand sanitizer versus handwashing: what are the planetary health consequences?

**DOI:** 10.1007/s11356-022-18918-4

**Published:** 2022-02-23

**Authors:** Brett Duane, Jessica Pilling, Sophie Saget, Paul Ashley, Allan R. Pinhas, Alexandra Lyne

**Affiliations:** 1 Department of Public Health, Trinity College Dublin, Lincoln Place, , Dublin, Ireland; 2 Department of Physics, University of Birmingham, Edgbaston, Birmingham, B15 2TT UK; 3 Department of Paediatric Dentistry, UCL Eastman Dental Institute, UCL, Gower Street, London, WC1E 6BT UK; 4 Department of Chemistry, University of Cincinnati, Cincinnati, OH USA

**Keywords:** Hand gel, Hand sanitizer, Planetary health, Environment, Carbon footprint, Planet, Industrial ecology

## Abstract

In order to reduce the transmission of pathogens, and COVID-19, WHO and NHS England recommend hand washing (HW) and/or the use of hand sanitizer (HS). The planetary health consequences of these different methods of hand hygiene have not been quantified. A comparative life cycle assessment (LCA) was carried out to compare the environmental impact of the UK population practising increased levels of hand hygiene during the COVID-19 pandemic for 1 year. Washing hands with soap and water was compared to using hand sanitizer (both ethanol and isopropanol based sanitizers were studied). The isopropanol-based HS had the lowest environmental impact in 14 out of the 16 impact categories used in this study. For climate change, hand hygiene using isopropanol HS produced the equivalent of 1060 million kg CO_2_, compared to 1460 million for ethanol HS, 2300 million for bar soap HW, and 4240 million for liquid soap HW. For both the ethanol and isopropanol HS, the active ingredient was the greatest overall contributing factor to the environmental impact (83.24% and 68.68% respectively). For HW with liquid soap and bar soap, there were additional contributing factors other than the soap itself: for example tap water use (28.12% and 48.68% respectively) and the laundering of a hand towel to dry the hands (10.17% and 17.92% respectively). All forms of hand hygiene have an environmental cost, and this needs to be weighed up against the health benefits of preventing disease transmission. When comparing hand sanitizers to handwashing with soap and water, this study found that using isopropanol based hand sanitizer is better for planetary health. However, no method of hand hygiene was ideal; isopropanol had a greater fossil fuel resource use than ethanol based hand sanitizer. More research is needed to find hand hygiene sources which do not diminish planetary health, and environmental impact is a consideration for public health campaigns around hand hygiene.

**Supplementary Information:**

The online version contains supplementary material available at 10.1007/s11356-022-18918-4.

## Introduction

Hand hygiene is one of the most important ways of avoiding pathogen transmission. (WHO 2020a).

COVID-19, the disease caused by a new coronavirus called SARS-CoV-2, was first reported to the World Health Organisation (WHO) on December 31^st^ 2019, originating in Wuhan, China. (WHO 2019) The disease was stated as a pandemic in 12 March 2020 (WHO 2020b). Currently (April 22nd, 2021), 141.8 million people have been infected, with 3.026 million deaths globally (ECDPC 2020).

To help prevent pathogen spread and in particular of Covid-19, the WHO recommends hand washing with soap and water (HW) or cleaning hands with alcohol hand sanitizer (HS) whenever hands are visibly dirty, as well as if hands are not visibly dirty. The WHO and Centre for Disease Control (CDC) both state that it is necessary to clean hands at key times with soap and water or HS with at least 60% alcohol. The WHO provides guidance for the contents of HS sanitizer based on ethanol or isopropanol (Centre for Disease Control Prevention 2020; WHO 2020c, d, e) The Coronavirus pandemic and subsequent public health recommendations have brought a shortage of HS all over the world (New York Times 2020).

The WHO has two recommended formulations for HS: either ethanol or isopropanol as the alcohol ‘active ingredient’, with glycerin as a moisturiser. The ethanol or isopropanol alcohol has been shown to inactivate SARS-COV-2 (Kratzel et al. 2020). Ethanol is produced as a bi-product of carbohydrate fermentation, whereas isopropanol alcohol (2-propanol) is manufactured by the indirect hydration of the fossil fuel propylene. (Yang 2007; Britannica 2020). Plain soap works by mechanical action to remove pathogens but also to inactivate enveloped viruses, such as the COVID-virus, dissolving the oily surface membrane (Sickbert-Bennett et al. 2005). Soap is made from reacting oil with a strong alkali or caustic (Hamner 2020).

Planetary health is the health of human civilization and the state of the natural systems on which it depends (Whitmee et al. 2015). The overall planetary (and public) health impact of hand hygiene is as yet unquantified. There are different ways to measure both environmental sustainability and the consequential planetary health impact. One method is service level carbon footprinting; this accounts for the climate change impact by looking at greenhouse gas emissions and global warming potential. However, not only is this a resource intensive process, but climate change and global warming is a single measure of sustainability and does not account for impacts such as eutrophication of water supplies, resource scarcity, and reduction in biodiversity, to name a few.

A life cycle assessment (LCA) can also be used to consider the entire life cycle of a product from ‘cradle to grave’. LCA data can be used to quantify multiple environmental impacts as well as other useful public health information such as Disability Adjusted Life Years (DALYs). DALYs consider both the time lost to premature death and reduced quality of life due to illness. (DALYs 2020).

Although LCAs have been used to compare different types of hand drying (Joseph et al. 2015), from our knowledge LCAs have not been used to compare the use of HS types compared with HW. The objectives of this study were to compare the environmental sustainability of the UK population using different methods of hand hygiene using ethanol and isopropanol HS vs using liquid and bar soap HW, in the context of the COVID-19 pandemic.

## Materials & methods

### Comparative LCA

A comparative attributional LCA of 4 different hand hygiene techniques was undertaken at the Eastman Dental Hospital, London, in partnership with the Dublin Dental University Hospital (Trinity College Dublin, Ireland). The four hand hygiene techniques were:

1. Ethanol-based HS: individual disinfects hands with 0.4 ml of hand sanitiser, made from WHO formulation I (WHO 2009)
2. Isopropanol-based HS: individual disinfects hands with 0.4 ml of hand sanitiser, made from WHO formulation II (WHO 2009)
3. Liquid soap HW: individual washes hands with 0.75 ml of liquid soap and 3.33L of tap water, drying hands with a laundered hand towel.
4. Bar soap HW: individual washes hands with 0.15 ml of bar soap and 3.33L of tap water, drying hands with a laundered hand towel.

**Fig. 1:**
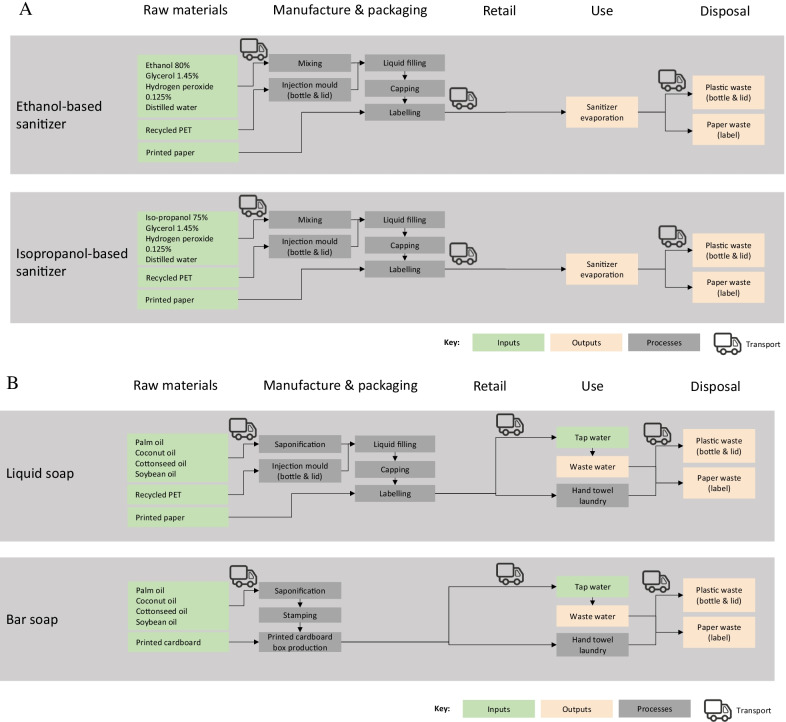
A System Boundaries for hand sanitizer, B System boundaries for handwashing

The functional unit was defined as the UK population practicing COVID-19-related hand hygiene for 1 year. This time period was chosen as it reflects the time that the COVID-19 pandemic is currently expected to be at least as prevalent. (Charumilind et al. 2020). The LCA methodology was applied in line with the ISO standards (ISO 2015) and PEF guidelines (European Commission Joint Research Centre 2018). The system boundaries are available in Fig. 1.

A life cycle inventory was created for each hand hygiene technique. Each technique was modelled as a generic product manufactured in the UK, not based upon a specific brand or product. The assumptions for each stage are detailed below. The life cycle inventory is available in Appendix B.

### Materials & manufacture

The components and manufacturing processes for hand sanitiser were based on the WHO hand hygiene guidelines (WHO, 2009). The weight of each constituent of the sanitiser was based on the density and volume needed to produce 1 L of product. Fragrances and pigments were excluded from all products as they were not consistent product by product, and were assumed to be present in equal quantities across all types of products assessed. It was assumed that components for hand sanitiser were mixed in a pressure-protected mixing machine and immediately packaged in screw top bottles to prevent evaporation, as per WHO guidelines (WHO, 2009). It was assumed that all liquid soap and sanitizer products were packaged by machine assembly in 1 L cylindrical plastic bottles (made from recycled polyethylene terephthalate) with a plastic lid (polyethylene) and a printed paper label that wraps around the bottle. The weight of the bottle, lid, and label were based on a tear down of a sample 1L sanitizer bottle. The constituents of the liquid and bar soap were assumed to include palm oil, cottonseed oil, and soybean oil as per the Ecoinvent (2020) dataset for a basic soap. For bar soap, it was assumed that 90 g bars of soap were stamped and packaged in printed cardboard boxes only, based on a tear down of a sample bar of soap.

### Transport

Assumptions for transport of the products from the factory to retailer, and then from retailer to consumer, were based on the PEF recommendations for modelling transport (European Commission Joint Research Centre 2018). This assumes the manufacturing location was 300 km from the UK retailer, i.e. a 600 km round via lorry (based on the weight of the transported products). The impact of retail processes themselves was excluded from the system boundaries of this LCA. The transport from the retailer to the consumer home was allocated transport was modelled following PEF guidelines (European Commission Joint Research Centre 2018).

### Consumer use

Based on hand hygiene advice to control the transmission of COVID-19 (NHS England 2019; DOHSC 2020), it was assumed that each individual in the UK would practice hand hygiene for COVID-19 purposes an average of 15 times per day. This is based on the average individual leaving the house twice daily (for work and one other occasion), using the toilet 5 times, coughing or sneezing twice, and preparing meals. The functional unit in this study was the entire UK population, which was assumed to be 66,796,800 individuals based on 2019 government data (Office for National Statistics 2020). The amount of sanitiser used per episode of hand hygiene was assumed to be 4 ml based on Zingg et al. (2016)^.^ The amount of soap used per episode of hand hygiene was deemed to be 0.35 g for the bar soap (McGill 2020) and 1.5 ml for liquid soap (based on the average volume an individual receives from 1 pump of a soap dispenser, Richter 2016).

Hand hygiene with HS does not require water. For HW with liquid or bar soap, it was assumed an individual would use room temperature tap water for an average of 40 s (NHS, 2019) with a UK tap using 5L of water per minute (DEFRA 2015a, b) (Table 1).

**Table 1:** Estimated consumer use per year per person

	Quantity per year per person	Water use
Sanitiser	21.9 l	-
Liquid soap	8.2 l	18,250 l
Bar soap	1.9 kg	18,250 l

Hand hygiene with HS does not require drying the hands with a towel as the contents evaporate. For HW with liquid or bar soap, the drying of hands with a hand towel was included. It was assumed individuals would be practicing HW at home, and therefore using a pre-existing hand towel to thoroughly dry their hands. Therefore, the manufacture of the towel was excluded from the system boundaries, but the laundering of the hand towel was included. It was assumed that each household would share a 350 g hand towel (based on an average of 2.3 people per household in the UK) and would launder this hand towel every other day (Sturt 2015; Statistica 2021; Chilton 2004).

### Disposal

For the bottled products, it was assumed that individuals would dispose of the plastic bottle and lid in plastic recycling, and the paper label in paper recycling in the UK. For the bar soap, it was assumed the cardboard box of soap was recycled.

For the ethanol HS, the amount of ethanol absorbed through the skin is negligible, and the amount of ethanol inhaled by the user is only a couple of percent (Brewer and Streel 2020). Thus, almost all of the ethanol in normal use (approximately 98%) is lost by evaporation into the air. Two percent of the ethanol was assumed to be excreted through the human body.

It is known that the ethanol in a hand rub sanitizer on a metal or glass plate at ambient temperature (approximately 21 degrees C) evaporates quite rapidly. The first-order half-life was found to be approximately 8 min, which means that half of the amount present at any time will evaporate in the next 8 min (Pinhas 2010). Therefore, it is assumed, given the warmer skin temperature (approximately 35 degrees C) and the thin film generated by rubbing of hands together, that almost all of the ethanol disappears by evaporation (the first-order half-life is estimated at less than 2 min).

For the isopropanol HS, the boiling point isopropyl alcohol is 82 degrees C and its vapor pressure at 35 degrees C is 95 mm Hg (NLM 2021). Unlike the measurements for ethanol in hand rub sanitizers, the kinetics of evaporation of isopropanol in hand rub sanitizers have not been measured. However, given these vapor pressure data, the rate of evaporation and the amount of evaporation at any time was assumed to be similar and the evaporation/metabolism in the human body was assumed to be the same. Glycerine and water were assumed to be absorbed by the human body and excreted through waste water.

### Data analysis

Data from the life cycle inventory was modelled and analysed in OpenLCA (2020) v11, alongside the reference database Ecoinvent v3.7.1. Appendix A describes the different midpoint impact categories and LCIA methods used in this study, based on the PEF guidance (European Commission Joint Research Centre 2018). The results were normalised against average global per capita reference values in order to compare the relative significance of each impact category. A contribution analysis was performed for the following endpoint impact categories; human health, ecosystem damage, and resource use. For liquid soap HW, a sensitivity analysis was performed to vary the quantity of soap and water used per episode of handwashing and examine the effect of the variation on the LCIA results. DALYs were calculated for each product using ReCiPe 2016 (H) Endpoint.

## Results

### Life cycle impact assessment

The results of the LCIA are shown in Table 2. The highlighted red cells represent the products with the highest impact in each category. HW with liquid soap and water had the greatest impact in 6 out of the 16 categories, followed by ethanol HS in 6 categories, and bar soap in 3 categories. Similarly, the highlighted green cells represent the products with the lowest impact in each category. Isopropanol HS had the lowest impact in 14 out of the 16 categories, followed by ethanol HS and bar soap (which had the lowest impact in fossil fuel use and photochemical ozone formation respectively).

**Table 2:** LCIA results

Impact category	LCIA results				
	Units	Sanitizer 1 (Ethanol based)	Sanitizer 2 (Isopropanol based)	Liquid Soap & water	Bar Soap & water
Climate change (CC)	kg CO_2_ eq	1.46E + 09	1.06E + 09	4.24E + 09	2.30E + 09
Acidification (FTA)	mol H + eq	1.94E + 07	4.47E + 06	1.85E + 07	9.38E + 06
Freshwater ecotoxicity (ECF)	CTU	2.19E + 10	9.90E + 08	1.35E + 10	7.02E + 09
Freshwater eutrophication (EUF)	kg P eq	4.29E + 05	1.44E + 05	9.28E + 06	2.65E + 06
Marine eutrophication (EUM)	kg N eq	1.01E + 07	9.94E + 05	1.54E + 07	5.62E + 06
Terrestrial eutrophication (EUT)	mol N eq	7.84E + 07	8.23E + 06	5.89E + 07	2.72E + 07
Carcinogenic effects (CE)	CTUh	6.99E + 01	1.60E + 01	2.61E + 02	3.07E + 02
Ionising radiation (IR)	kg U^235^ eq	9.05E + 07	6.08E + 07	2.91E + 08	9.92E + 07
Non carcinogenic effects (NCE)	CTUh	1.64E + 03	1.34E + 02	4.65E + 02	4.89E + 02
Ozone layer depletion (OD)	kg CFC^11^ eq	1.66E + 02	6.48E + 01	2.68E + 02	1.71E + 02
Photochemical ozone creation (POF)	kg NMVOC eq	6.43E + 08	1.39E + 08	1.15E + 07	6.82E + 06
Respiratory inorganics (RI)	Disease inc	1.41E + 02	3.99E + 01	2.72E + 02	1.48E + 02
Water use (DW)	m^3^ water eq	6.26E + 09	3.59E + 08	1.14E + 10	1.19E + 10
Fossil fuel use (FF)	MJ	1.58E + 10	2.82E + 10	3.57E + 10	2.60E + 10
Land use (LU)	Points	2.47E + 11	1.06E + 10	2.38E + 11	7.37E + 10
Mineral/ metal use (MM)	kg Sb eq	9.27E + 03	7.24E + 03	3.06E + 04	1.94E + 04

### Normalised results and contribution analysis

**Fig. 2:**
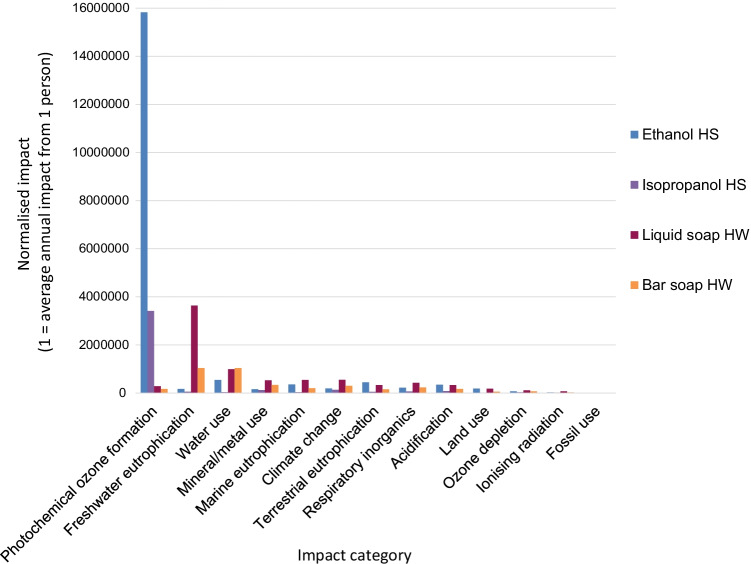
Normalised LCIA results for different types of hand hygiene

The normalised results compare the impact of using HS or HW against one global person’s annual share of all emission and resource use in the world (the impact that the “average Joe” would be expected to make from living their daily lives for 1 year). Figure 2 presents the normalised results. An average person would use “1” per year in each category. Each impact category has a different normalisation factor, which is part of the LCIA methods. These factors are discussed in Appendix A.

As per PEF recommendations, the toxicity categories have been removed (European Commission Joint Research Centre 2018). Photochemical ozone formation had the greatest normalised impact for both types of HS, equivalent to the annual contribution of 16 million people for ethanol HS and 3 million people for isopropanol HS. Freshwater eutrophication was the most significant impact category for both types of HW, equivalent to the annual contribution of 3.5 million people for the liquid soap and 1 million people for the bar soap.

**Fig. 3:**
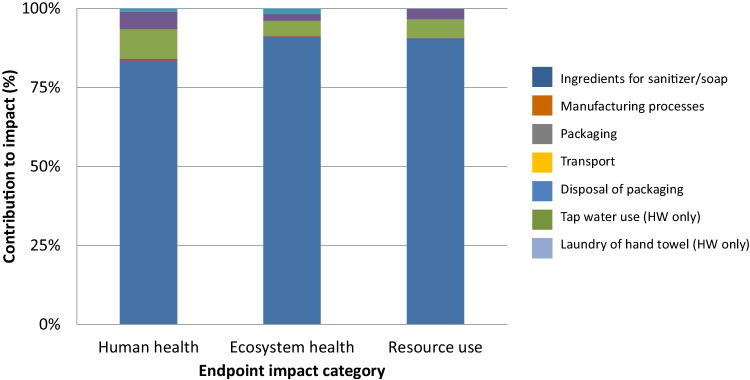
Contribution analysis for ethanol-based HS

**Fig. 4:**
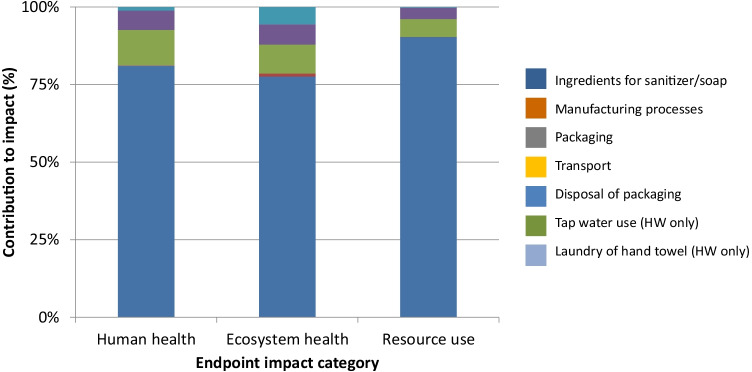
Contribution analysis for isopropanol-based HS

**Fig. 5:**
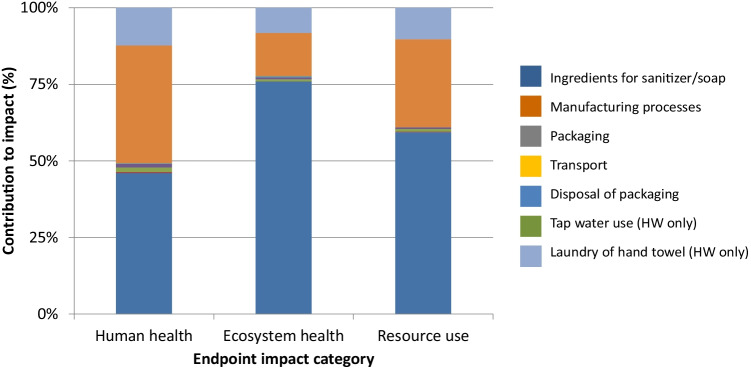
Contribution analysis for liquid soap HW

**Fig. 6:**
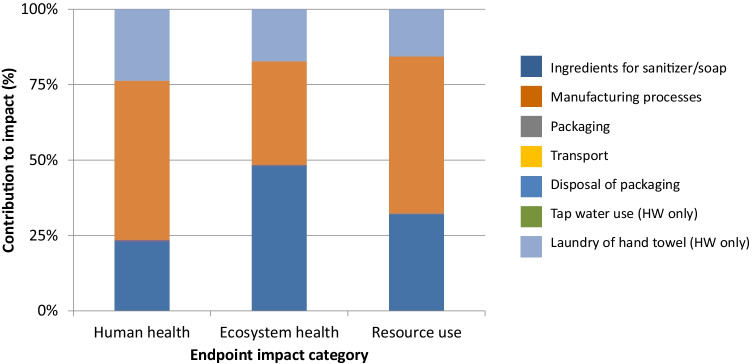
Contribution analysis for bar soap HW

A contribution analysis was carried out for each impact category. Figures 3, 4, 5, and 6 show the contributions for each type of hand hygiene to each endpoint impact category. For both types of HS, it was the ingredients to make the sanitizer that had the greatest contribution (83.46–91.04% for ethanol HS, and 77.59–90.29% for isopropanol HS). When broken down further into the individual ingredients, the active ingredient in the HS was the main contributor, and the remaining ingredients (glycerine, water, and hydrogen peroxide) did not contribute more than 13% in any category. The packaging (a plastic bottle and paper label) contributed 4.80–9.46% for the ethanol HS and 5.76–11.39% for the isopropanol HS. For the liquid soap HW, the greatest contributor was the soap itself (46.04–75.92%) followed by tap water use (14.11–38.51%) and laundry for the hand towel (8.25–12.32%). For the bar soap HW, the greatest contributor was the tap water (34.42–52.86%) followed by the soap (22.88–47.99%) and laundry for the hand towel (17.25–23.68%). For both soaps, the packaging contributed no more than 1.5% in any category.

### Sensitivity analysis

A sensitivity analysis for liquid soap HW was performed to examine the effect of increasing or decreasing the volume of liquid soap, as different brands will dispense different amounts of soap per ‘pump’. The original assumption was that the pump would dispense 1.5 ml of liquid soap. Halving the pump volume, to 0.75 ml, reduced the LCIA results by an average of 31% (range from 13% in carcinogenic effects to 47% in freshwater eutrophication). Similarly, doubling the pump volume, to 3 ml, increased the LCIA results by an average of 61% (range from 26% in carcinogenic effects to 94% in freshwater eutrophication).

Another sensitivity analysis for liquid soap HW was performed to examine the effect of increasing or decreasing the volume of water used to wash the hands. The original assumption was that 3.33L of water were used per episode of handwashing (tap running for 40 s at 5L/min). Halving the water use, to 1.67L, reduced the LCIA impact by an average of 86% (range from 66% in carcinogenic effects to 98% in land use); and doubling the water use, to 6.66L, increased the LCIA impact by an average of 28% (range from 3% in land use to 69% in carcinogenic effects).

### DALYs

**Fig. 7:**
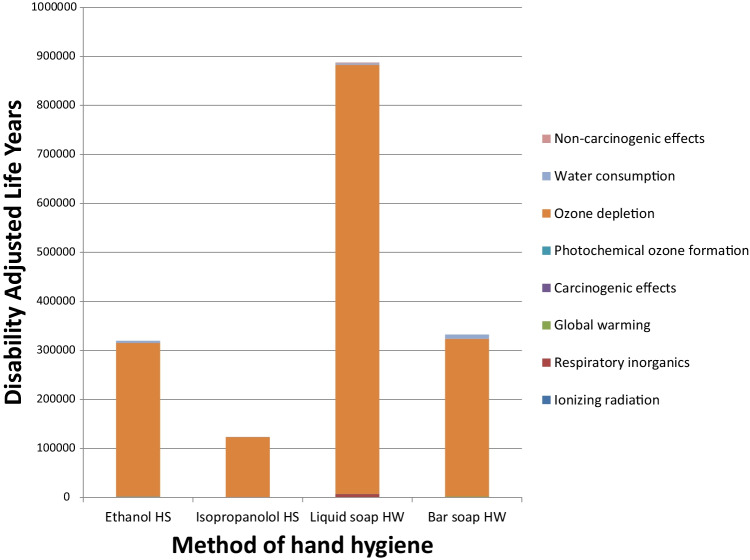
DALY contributions for each type of hand hygiene

The results of the DALY impact calculations are shown in Fig. 7. The functional unit was the entire UK population practising hand hygiene for 1 year: when adjusted to per person DALY, this impact was equivalent to 114 h for liquid soap HW, compared to 43 h for bar soap HW, 41 h for ethanol-based HS and just 16 h for the isopropanol-based HS. The biggest contributor to the DALY impact for all methods of hand hygiene was ozone depletion (contributing 97–99% of the total DALY impact).

## Discussion

Hoekman et al. (2018) provides a recent review of the environmental concerns associated with hand sanitiser. Our paper has developed this concept further by performing a Life Cycle Assessment of different methods of hand hygiene with santizer and soap. Our results demonstrate that, from an environmental perspective, there is a clear advantage of using isopropanol-based HS instead of other methods, such as ethanol-based HS and HW with liquid or bar soap. From a climate change perspective, the impact of ethanol-based sanitizer was 1.38 times greater than isopropanol sanitizer, the impact of bar soap was 2.2 times greater, and the impact of liquid soap was 4 times greater. In fact, isopropanol HS performed better than the other methods of hand hygiene in 14 out of the 16 measures of sustainability. It was only outperformed in fossil use and photochemical ozone formation.

The overall human health impact was measured using DALYs. The biggest contributor to the DALY impact, for all methods of hand hygiene, was ozone depletion, which damages the protective ozone layer surrounding our planet. The next highest contributor was for the use of water in each product. Water was required at different stages of the pathway. The first was in packaging and manufacture, water is required to grow and convert the fruit for the ethanol gel, manufacture the isopropanol and also to mould the plastic associated with the packaging. It is also required to manufacture the soap. It is not possible to eliminate water from the manufacturing process, however it may be possible for industry to consider more sustainable packaging which doesn’t have such a need for this high water consumption. The other major use of water was in the actual handwashing process. For HW with any type of soap, water is used to wash hands, and undertake laundry processes. Water is also required to treat the waste water that is produced from this process. This is discussed further below.

There is some evidence that one type of HS might be more clinically effective than another. One study showed that 80% ethanol HS, was more effective than isopropanol (Hübner et al. 2006). Another clinical study showed that ethanol based disinfectant had reduced effectiveness compared to antiseptic handwashing in saline (Hoekman et al. 2018). The type of debris on the hands might also be a factor in the effectiveness of both products. Neither of the soaps used in this study were antiseptic, as WHO and CDC guidelines do not recommend antiseptic hand washing for population level use.

Neither of these studies examined clinical effectiveness against COVID-19 transmission, and so, this discussion will focus on the environmental impact of the four hand hygiene methods.

### Soap

The use of soap is an important part of hand hygiene with one study showing handwashing with water alone reduced the presence of bacteria to 23% (*p* < 0.001) but using additional plain soap and water reduced the presence of bacteria to a lower 8% (comparison of both handwashing arms: *p* < 0.001) (Burton et al. 2011). The benefit of washing with soap was also confirmed in a study by Luby et al. (2011) where there was a marginal but statistically significant difference between bacterial counts using soap and not using soap.

The CDC recommends either bar soap or liquid soap (Centre for Disease Control Prevention 2020). Within the Ecoinvent database only one category of soap exists, and this was used to model both liquid and bar soap. Therefore, this paper then only looks at the differences in weights of the actual soap, and the reduced packaging needed for bar soap compared with liquid soap.

The LCA for liquid soap showed that the soap oils (palm oil, cottonseed oil, soybean oil, coconut oil) had a contribution of between 24% (toward the ionizing radiation impact) and 93.60% (towards the freshwater eutrophication aspect) of the environmental footprint. Freshwater eutrophication impact arises from a number of sources, including growing the oils to produce the soap (e.g. the growing and processing of palm oil) as well as water required to manufacture the soap. It is acknowledged that there is wide variability within the constituents of soap. We know that palm oil is associated with high negative impacts on the environment, but there is no evidence to suggest alternatives would be any better (Standard 2020). Palm oil has been blamed for deforestation, peatland draining and burning in SE Asia, but there is been little research into the impact and trade-off of other comparable product.(Meijaard et al. 2020) In essence if the planet needs to grow the product, it will have some environmental implications, more research is needed to be able to produce soap whilst lowering these factors.

For both liquid and bar soap, the process of handwashing requires using tap water (tap water use contributed 28%). There are a number of ways to reduce this footprint. Washing your hands for less time, or with less soap (halving the pump volume reduced LCIA results by of 31%) would be one factor, but might also reduce the effectiveness of the process. The NHS (2019) suggests you should use room temperature tap water for an average of 40 s (NHS, 2019) with a UK tap using 5 l of water per minute (DEFRA 2015a, b). This is different from the advice of the CDC which suggests you should wash your hands for at least 20 s. The evidence for how much time you should be spending washing your hands is poor. There are a limited number of studies, with one problem being that simply reducing bacteria doesn’t necessarily equate to better hand hygiene or personal health (Luby et al. 2007). The amount of time depends on the type and quantity of debris/pathogen on the hands, but there is evidence to suggest that 15–30 s is more beneficial for pathogen removal than washing for a shorter time period (Fuls et al. 2008; Jensen et al. 2015). There is also no evidence to suggest the temperature of the water is relevant when it comes to reduction in pathogens in HW (Michaels et al. 2002; Laestadius and Dimberg 2005; Carrico et al. 2013).

The laundry of the hand towel contributed an average of 10.17% for liquid soap and 17.92% for hand soap. If the consumer used a smaller hand towel, or washed their towels weekly instead of every second day, this contribution would lower. The Ecoinvent dataset for laundry includes the cleaning, drying and ironing of laundry therefore removing the machine drying and ironing of your towels could also reduce the overall environmental impact (Benjamen 2020).

Our study reinforces the environmental benefits of using bar soap compared with liquid soap. This is for a number of reasons including less plastic packaging with the latter, but also as less soap is released when using a bar. It is unsure as to whether this reduction in soap may also reduce pathogenic efficacy of the bar.

### Hand sanitizer

Rubbing most commercial hand sanitizers on one’s hands for as little as 15 s reduces the amount of many viruses by a factor of 1000 and reduces the amount of a wide range of bacteria by 100,000 (Golin et al. 2020). HS can contain either isopropanol or ethanol. From an environmental perspective, the ethanol within the hand sanitiser is known to be toxic(Von Blottnitz and Curran 2007). Evaporation of ethanol, causes environmental problems, such as increased ethanol found in surface and ground water and a change in photochemical ozone concentration, and the resultant summer smog has been shown with bioethanol fuel. (Jacobson 2007). Isopropanol spills are less problematic, as it breaks down rapidly into organic compounds, but as Mahmood et al. (2020) explains, the substance will still deplete oxygen in a water body.

Within the impact analysis, isopropanol performed better across nearly all measurements compared with ethanol, except for fossil fuel use (isopropanol is made from fossil fuels, whereas the ethanol in this study was made via fermentation). When the results were normalised, fossil fuel use was found to be the least important of the 16 impact categories measured in this LCA (the normalised results for fossil fuel use was 0 for all products). The fact that the impact of ethanol via fermentation was worse for the environment than a fossil fuel based isopropanol is not as surprising as it seems, and is in line with fuel studies showing the problems with using ethanol as a fossil fuel substitute. Northern American studies are mostly based on ethanol produced from corn. A mono-culture crop such as corn can inevitably reduce plant and animal biodiversity, and contribute to erosion, nutrient run off and other adverse environmental impacts. Corn ethanol production uses a modest amount of water which can be a significant resource problem where there are constraints of water (Hoekman et al. 2018; Hoekman and Broch 2018, United States 2020). The LCA process draws on a British ethanol which is produced mostly from maize, sugar cane and rye (Ecoinvent dataset v3.7). Each of these require significant planetary resources to grow the product with less resource required to convert the product into the hand gel. The impacts of sugar cane (of which a significant portion of bioethanol are derived) have been recently reviewed and are similar to those of corn (El Chami et al. 2020).

Using less hand sanitizer would reduce its environmental impact but potentially also reduce its ability to disinfect effectively. Zingg et al (2016) found that 3 ml of HS was not always enough to disinfect larger hand sizes and thoroughly cover both sides of the hands before drying.

In this study, hand sanitizer was in general more environmentally sustainable than handwashing with liquid soap, most likely because handwashing with soap and water involves water use and drying the hands with a hand towel, which hand gels do not.

## Conclusion

Hand hygiene is recommended by WHO, CDC, and NHS England to prevent the spread of COVID-19. Hand hygiene using hand sanitizer or soap and water all have an environmental impact, hand sanitizer was more environmentally sustainable than handwashing with soap. Although hand hygiene is a substantial tool in the public health armamentarium, it does cause significant planetary harm. More research is needed to create hand gels which are more environmentally friendly.

## Supplementary Information

Below is the link to the electronic supplementary material.

Supplementary file1 (DOCX 33 KB)

Supplementary file2 (PDF 0.99 MB)

## Data Availability

The datasets generated and/or analysed during the current study are available in the tables in the document. The raw material inputted is shown in Appendix B.
