# Supplementary material for: Hand hygiene with hand sanitizer versus handwashing: what are the planetary health consequences?
Source: Environ Sci Pollut Res Int. 2022 Feb 23;29(32):48736–47. doi: 10.1007/s11356-022-18918-4 (PMC8865176; doi:10.1007/s11356-022-18918-4)
Supplement: Supplementary file 1 — Supplementary file1 (DOCX 33 KB) [file 11356_2022_18918_MOESM1_ESM.docx]

# Appendix A: Impact categories and LCIA methods used in this study

| **Impact category (abbreviation)** | **LCIA method (units)** | **Description** |
| --- | --- | --- |
| Climate change (CC) | IPCC 2013 GWP 100a (kg CO2 eq) | Potential for global warming from greenhouse gas emissions. |
| Ecosystem quality: Freshwater and terrestrial acidification (FTA) | ILCD 2011 Midpoint+ (Mol H+ eq) | Acidification of soils and freshwater due to gas release. |
| Ecosystem quality: ecotoxicity freshwater (ECF) | ILCD 2011 Midpoint+ (CTUe) | Harmful effects of toxic substances on freshwater organisms. |
| Ecosystem quality: eutrophication freshwater (EUF) | ILCD 2011 Midpoint+ (kg P eq) | Changes in freshwater organisms and ecosystems caused by excess nutrients. |
| Ecosystem quality: eutrophication marine (EUM) | ILCD 2011 Midpoint + (kg N eq) | Changes in marine organisms and ecosystems caused by excess nutrients. |
| Ecosystem quality: eutrophication terrestrial (EUT) | ILCD 2011 Midpoint + (Molc N eq) | Changes in land organisms from excess nutrients in soil and air. |
| Human health: cancer effects (CE) | ILCD 2011 Midpoint+ (CTUh) | Harm to human health that causes or increases cancer risk. |
| Human health: ionizing radiation (IR) | ILCD 2011 Midpoint + (kBq U-235 eq) | Potential damage to human DNA from ionizing radiation. |
| Human Health: non-cancer effects (NCE) | ILCD 2011 Midpoint+ (CTUh) | Harm to human health that is not related to cancer or ionising radiation. |
| Human health: respiratory inorganics (RI) | PM method (Disease inc.) | Harm to human health caused by particulate matter emissions (respiratory disease). |
| Human health: photochemical ozone formation (POF) | ILCD 2011 Midpoint + (kg NMVOC eq) | Harm to human health from gas emissions that contribute to smog in the lower atmosphere. |
| Resource use: Land use (LU) | Soil quality index based on LANCA (Pt) | Depletion of natural resources, change in soil quality, and reduction in biodiversity. |
| Human health: Ozone depletion (OD) | ILCD 2011 Midpoint+ (kg CFC11 eq) | Air emissions causing stratospheric ozone layer destruction. |
| Resource use: fossils FF) | CML-IA baseline (MJ) | Depletion of natural fossil fuels. |
| Resource use: minerals and metals (MM) | CML-IA baseline (kg Sb eq) | Depletion of natural non-fossil fuel resources. |
| Resource use: dissipated water (DW) | AWARE (m3 depriv.) | Potential for water deprivation to humans and ecosystems globally. |

# Appendix B: Life cycle inventory for each type of hand hygiene

| **Bar soap inputs** | **Amount** | **Unit** | **Provider** |
| --- | --- | --- | --- |
| Carton board box production, with offset printing | 979865.8468 | Kg | Market for carton board box production, with offset printing \| carton board box production, with offset printing \| Cutoff, U - GLO |
| Electricity, medium voltage | 11079.52886 | Kwh | Market for electricity, medium voltage \| electricity, medium voltage \| Cutoff, U - GB |
| Soap | 1.28E+08 | Kg | Market for soap \| soap \| Cutoff, U - GLO |
| Tap water | 1.22E+12 | Kg | Market for tap water \| tap water \| Cutoff, U - Europe without Switzerland |
| Transport, freight, lorry 3.5-7.5 metric ton, EURO3 | 3.23E+07 | Kg*km | Market for transport, freight, lorry 3.5-7.5 metric ton, EURO3 \| transport, freight, lorry 3.5-7.5 metric ton, EURO3 \| Cutoff, U - RER |
| Transport, freight, lorry >32 metric ton, EURO4 | 7.74E+10 | Kg*km | Market for transport, freight, lorry >32 metric ton, EURO4 \| transport, freight, lorry >32 metric ton, EURO4 \| Cutoff, U - RER |
| Transport, passenger car | 42213.47248 | Km | Market for transport, passenger car \| transport, passenger car \| Cutoff, U - RER |
| Washing, drying and finishing laundry | 1.86E+09 | Kg | Market for washing, drying and finishing laundry \| washing, drying and finishing laundry \| Cutoff, U - GLO |
| **Bar soap outputs** |  |  |  |
| Waste paperboard | 979865.8468 | Kg | Market for waste paperboard \| waste paperboard \| Cutoff, U - GB |
| Wastewater, unpolluted, from residence | 1.22E+12 | L | Market for wastewater, unpolluted, from residence \| wastewater, unpolluted, from residence \| Cutoff, U - row |
| **Hand gel 1 inputs** |  |  |  |
| Ethanol, without water, in 99.7% solution state, from fermentation | 9.62E+08 | Kg | Market for ethanol, without water, in 99.7% solution state, from fermentation \| ethanol, without water, in 99.7% solution state, from fermentation \| Cutoff, U - GLO |
| Glycerine | 2.68E+07 | Kg | Market for glycerine \| glycerine \| Cutoff, U - RER |
| Hydrogen peroxide, without water, in 50% solution state | 3651569.712 | Kg | Market for hydrogen peroxide, without water, in 50% solution state \| hydrogen peroxide, without water, in 50% solution state \| Cutoff, U - RER |
| Water, deionised | 2.18E+08 | Kg | Market for water, deionised \| water, deionised \| Cutoff, U - Europe without Switzerland |
| Electricity, medium voltage | 1463851.872 | Kwh | Market for electricity, medium voltage \| electricity, medium voltage \| Cutoff, U - GB |
| Electricity, medium voltage | 1829814.84 | Kwh | Market for electricity, medium voltage \| electricity, medium voltage \| Cutoff, U - GB |
| Electricity, medium voltage | 426956.796 | Kwh | Electricity voltage transformation from high to medium voltage \| electricity, medium voltage \| Cutoff, U - GB |
| Injection moulding | 6.29E+07 | Kg | Injection moulding \| injection moulding \| Cutoff, U - RER |
| Polyethylene terephthalate, granulate, bottle grade, recycled | 5.71E+07 | Kg | Market for polyethylene terephthalate, granulate, bottle grade, recycled \| polyethylene terephthalate, granulate, bottle grade, recycled \| Cutoff, U - row |
| Polyethylene, high density, granulate | 5855407.488 | Kg | Market for polyethylene, high density, granulate \| polyethylene, high density, granulate \| Cutoff, U - GLO |
| Electricity, medium voltage | 406625.52 | Kwh | Market for electricity, medium voltage \| electricity, medium voltage \| Cutoff, U - GB |
| Printed paper, offset | 182981.484 | Kg | Market for printed paper, offset \| printed paper, offset \| Cutoff, U - GLO |
| Transport, freight, lorry 3.5-7.5 metric ton, EURO3 | 3.19E+08 | Kg*km | Market for transport, freight, lorry 3.5-7.5 metric ton, EURO3 \| transport, freight, lorry 3.5-7.5 metric ton, EURO3 \| Cutoff, U - RER |
| Transport, freight, lorry >32 metric ton, EURO4 | 7.65E+11 | Kg*km | Market for transport, freight, lorry >32 metric ton, EURO4 \| transport, freight, lorry >32 metric ton, EURO4 \| Cutoff, U - RER |
| Transport, passenger car | 2.81E+07 | Km | Market for transport, passenger car \| transport, passenger car \| Cutoff, U - RER |
| **Hand gel 1 outputs** |  |  |  |
| Ethanol | 9.43E+08 | Kg | N/A: elementary flow |
| Ethanol | 1.92E+07 | Kg | N/A: elementary flow |
| Hydrogen peroxide | 3578540 | Kg | N/A: elementary flow |
| Hydrogen peroxide | 73031.42857 | Kg | N/A: elementary flow |
| Waste paperboard | 182981.484 | Kg | Market for waste paperboard \| waste paperboard \| Cutoff, U - GB |
| Waste polyethylene | 5855407.488 | Kg | Market for waste polyethylene \| waste polyethylene \| Cutoff, U - GB |
| Waste polyethylene terephthalate | 5.71E+07 | Kg | Market for waste polyethylene terephthalate \| waste polyethylene terephthalate \| Cutoff, U - GB |
| Wastewater, from residence | 2.45E+08 | L | Market for wastewater, from residence \| wastewater, from residence \| Cutoff, U - row |
| **Hand gel 2 inputs** |  |  |  |
| Glycerine | 2.68E+07 | Kg | Market for glycerine \| glycerine \| Cutoff, U - RER |
| Hydrogen peroxide, without water, in 50% solution state | 3651569.712 | Kg | Market for hydrogen peroxide, without water, in 50% solution state \| hydrogen peroxide, without water, in 50% solution state \| Cutoff, U - RER |
| Isopropanol | 8.65E+08 | Kg | Market for isopropanol \| isopropanol \| Cutoff, U - RER |
| Water, deionised | 3.38E+08 | Kg | Market for water, deionised \| water, deionised \| Cutoff, U - Europe without Switzerland |
| Electricity, medium voltage | 1463851.872 | Kwh | Market for electricity, medium voltage \| electricity, medium voltage \| Cutoff, U - GB |
| Electricity, medium voltage | 1829814.84 | Kwh | Market for electricity, medium voltage \| electricity, medium voltage \| Cutoff, U - GB |
| Electricity, medium voltage | 426956.796 | Kwh | Market for electricity, medium voltage \| electricity, medium voltage \| Cutoff, U - GB |
| Injection moulding | 6.29E+07 | Kg | Injection moulding \| injection moulding \| Cutoff, U - RER |
| Polyethylene terephthalate, granulate, bottle grade, recycled | 5.71E+07 | Kg | Market for polyethylene terephthalate, granulate, bottle grade, recycled \| polyethylene terephthalate, granulate, bottle grade, recycled \| Cutoff, U - row |
| Polyethylene, high density, granulate | 5855407.488 | Kg | Polyethylene, high density, granulate, recycled to generic market for high density PE granulate \| polyethylene, high density, granulate \| Cutoff, U - Europe without Switzerland |
| Electricity, medium voltage | 406625.52 | Kwh | Market for electricity, medium voltage \| electricity, medium voltage \| Cutoff, U - GB |
| Printed paper, offset | 182981.484 | Kg | Market for printed paper, offset \| printed paper, offset \| Cutoff, U - GLO |
| Transport, freight, lorry 3.5-7.5 metric ton, EURO3 | 3.24E+08 | Kg*km | Market for transport, freight, lorry 3.5-7.5 metric ton, EURO3 \| transport, freight, lorry 3.5-7.5 metric ton, EURO3 \| Cutoff, U - RER |
| Transport, freight, lorry >32 metric ton, EURO4 | 7.78E+11 | Kg*km | Market for transport, freight, lorry >32 metric ton, EURO4 \| transport, freight, lorry >32 metric ton, EURO4 \| Cutoff, U - RER |
| Transport, passenger car | 2.81E+07 | Km | Market for transport, passenger car \| transport, passenger car \| Cutoff, U - RER |
| **Hand gel 2 input** |  |  |  |
| 2-Propanol (Elementary flows/Emission to air/high population density) | 8.47E+08 | Kg | N/A: elementary flow |
| 2-Propanol (Elementary flows/Emission to water/unspecified) | 1.73E+07 | Kg | N/A: elementary flow |
| Hydrogen peroxide (Elementary flows/Emission to air/unspecified) | 3580000 | Kg | N/A: elementary flow |
| Hydrogen peroxide (Elementary flows/Emission to water/unspecified) | 73000 | Kg | N/A: elementary flow |
| Waste paperboard | 182981.484 | Kg | Market for waste paperboard \| waste paperboard \| Cutoff, U - GB |
| Waste polyethylene | 5855407.488 | Kg | Market for waste polyethylene \| waste polyethylene \| Cutoff, U - GB |
| Waste polyethylene terephthalate | 5.71E+07 | Kg | Market for waste polyethylene terephthalate \| waste polyethylene terephthalate \| Cutoff, U - GB |
| Wastewater, from residence | 3.65E+08 | L | Market for wastewater, from residence \| wastewater, from residence \| Cutoff, U - row |
| **Liquid soap inputs** |  |  |  |
| Soap | 4.94E+08 | Kg | Market for soap \| soap \| Cutoff, U - GLO |
| Electricity, medium voltage | 686180.565 | Kwh | Market for electricity, medium voltage \| electricity, medium voltage \| Cutoff, U - GB |
| Electricity, medium voltage | 160108.7985 | Kwh | Market for electricity, medium voltage \| electricity, medium voltage \| Cutoff, U - GB |
| Injection moulding | 2.36E+07 | Kg | Injection moulding \| injection moulding \| Cutoff, U - RER |
| Polyethylene terephthalate, granulate, bottle grade, recycled | 2.14E+07 | Kg | Market for polyethylene terephthalate, granulate, bottle grade, recycled \| polyethylene terephthalate, granulate, bottle grade, recycled \| Cutoff, U - row |
| Polyethylene, high density, granulate | 2195777.808 | Kg | Polyethylene, high density, granulate, recycled to generic market for high density PE granulate \| polyethylene, high density, granulate \| Cutoff, U - Europe without Switzerland |
| Electricity, medium voltage | 152484.57 | Kwh | Market for electricity, medium voltage \| electricity, medium voltage \| Cutoff, U - GB |
| Printed paper, offset | 68618.0565 | Kg | Market for printed paper, offset/printed paper, offset/Cutoff, U-GLO |
| Tap water | 1.22E+12 | Kg | Market for tap water \| tap water \| Cutoff, U - Europe without Switzerland |
| Washing, drying and finishing laundry | 1.86E+09 | Kg | Washing, drying and finishing laundry \| washing, drying and finishing laundry \| Cutoff, U - GLO |
| Transport, freight, lorry 3.5-7.5 metric ton, EURO3 | 1.29E+08 | Kg*km | Market for transport, freight, lorry 3.5-7.5 metric ton, EURO3 \| transport, freight, lorry 3.5-7.5 metric ton, EURO3 \| Cutoff, U - RER |
| Transport, freight, lorry >32 metric ton, EURO4 | 3.11E+11 | Kg*km | Market for transport, freight, lorry >32 metric ton, EURO4 \| transport, freight, lorry >32 metric ton, EURO4 \| Cutoff, U - RER |
| Transport, passenger car | 1.05E+07 | Km | Market for transport, passenger car \| transport, passenger car \| Cutoff, U - RER |
| **Liquid soap outputs** |  |  |  |
| Wastewater, unpolluted, from residence | 1.22E+12 | L | Market for wastewater, unpolluted, from residence \| wastewater, unpolluted, from residence \| Cutoff, U - row |
| Waste paperboard | 68618.0565 | Kg | Market for waste paperboard \| waste paperboard \| Cutoff, U - GB |
| Waste polyethylene | 2195777.808 | Kg | Market for waste polyethylene \| waste polyethylene \| Cutoff, U - GB |
| Waste polyethylene terephthalate | 2.14E+07 | Kg | Market for waste polyethylene terephthalate \| waste polyethylene terephthalate \| Cutoff, U - GB |
