# Supplementary material for: Hand hygiene with hand sanitizer versus handwashing: what are the planetary health consequences?
Source: Environ Sci Pollut Res Int. 2022 Feb 23;29(32):48736–47. doi: 10.1007/s11356-022-18918-4 (PMC8865176; doi:10.1007/s11356-022-18918-4)
Supplement: Supplementary file 2 — Supplementary file2 (PDF 0.99 MB) [file 11356_2022_18918_MOESM2_ESM.pdf]

Appendix A: Impact categories and LCIA methods used in this study

Impact category (abbreviation)	LCIA method (units)	Description
Climate change (CC)	IPCC 2013 GWP 100a (kg CO2 eq)	Potential for global warming from greenhouse gas emissions.
Ecosystem quality: Freshwater and terrestrial acidification (FTA)	ILCD 2011 Midpoint+ (Mol H+ eq)	Acidification of soils and freshwater due to gas release.
Ecosystem quality: ecotoxicity freshwater (ECF)	ILCD 2011 Midpoint+ (CTUe)	Harmful effects of toxic substances on freshwater organisms.
Ecosystem quality: eutrophication freshwater (EUF)	ILCD 2011 Midpoint+ (kg P eq)	Changes in freshwater organisms and ecosystems caused by excess nutrients.
Ecosystem quality: eutrophication marine (EUM)	ILCD 2011 Midpoint + (kg N eq)	Changes in marine organisms and ecosystems caused by excess nutrients.
Ecosystem quality: eutrophication terrestrial (EUT)	ILCD 2011 Midpoint + (Molc N eq)	Changes in land organisms from excess nutrients in soil and air.
Human health: cancer effects (CE)	ILCD 2011 Midpoint+ (CTUh)	Harm to human health that causes or increases cancer risk.
Human health: ionizing radiation (IR)	ILCD 2011 Midpoint + (kBq U-235 eq)	Potential damage to human DNA from ionizing radiation.
Human Health: non-cancer effects (NCE)	ILCD 2011 Midpoint+ (CTUh)	Harm to human health that is not related to cancer or ionising radiation.

Human health: respiratory inorganics (RI)	PM method (Disease inc.)	Harm to human health caused by particulate matter emissions (respiratory disease).
Human health: photochemical ozone formation (POF)	ILCD 2011 Midpoint + (kg NMVOC eq)	Harm to human health from gas emissions that contribute to smog in the lower atmosphere.
Resource use: Land use (LU)	Soil quality index based on LANCA (Pt)	Depletion of natural resources, change in soil quality, and reduction in biodiversity.
Human health: Ozone depletion (OD)	ILCD 2011 Midpoint+ (kg CFC11 eq)	Air emissions causing stratospheric ozone layer destruction.
Resource use: fossils FF)	CML-IA baseline (MJ)	Depletion of natural fossil fuels.
Resource use: minerals and metals (MM)	CML-IA baseline (kg Sb eq)	Depletion of natural non-fossil fuel resources.
Resource use: dissipated water (DW)	AWARE (m3 depriv.)	Potential for water deprivation to humans and ecosystems globally.

Impact category	Unit	Normalization factor
Acidification	mol H+ eq	55.55555556
Climate change	kg CO2 eq	8097.165992
Ecotoxicity, freshwater	CTUe	42680.32437
Eutrophication, freshwater	kg P eq	1.606941989
Eutrophication, marine	kg N eq	19.54652072
Eutrophication, terrestrial	mol N eq	176.7408978
Human toxicity, cancer	CTUh	1.69E-05
Human toxicity, non-cancer	CTUh	2.30E-04
Ionising radiation	kBq U-235 eq	4219.409283
Land use	Pt	819672.1311
Ozone depletion	kg CFC11 eq	0.053648069
Particulate matter	disease inc.	5.95E-04
Photochemical ozone formation	kg NMVOC eq	40.60089322
Resource use, fossils	MJ	65019.50585
Resource use, minerals and metals	kg Sb eq	0.063653724
Water use	m3 depriv.	11469.20518

Appendix B: Life cycle inventory for each type of hand hygiene

**Bar soap inputs**	**Amount**	**Unit**	**Provider**
Carton board box production, with offset printing	979865.8468	Kg	Market for carton board box production, with offset printing | carton board box production, with offset printing | Cutoff, U - GLO
Electricity, medium voltage	11079.52886	Kwh	Market for electricity, medium voltage | electricity, medium voltage | Cutoff, U - GB
Soap	1.28E+08	Kg	Market for soap | soap | Cutoff, U - GLO
Tap water	1.22E+12	Kg	Market for tap water | tap water | Cutoff, U - Europe without Switzerland
Transport, freight, lorry 3.5-7.5 metric ton, EURO3	3.23E+07	Kg*km	Market for transport, freight, lorry 3.5-7.5 metric ton, EURO3 | transport, freight, lorry 3.5-7.5 metric ton, EURO3 | Cutoff, U - RER
Transport, freight, lorry >32 metric ton, EURO4	7.74E+10	Kg*km	Market for transport, freight, lorry >32 metric ton, EURO4 | transport, freight, lorry >32 metric ton, EURO4 | Cutoff, U - RER
Transport, passenger car	42213.47248	Km	Market for transport, passenger car | transport, passenger car | Cutoff, U - RER
Washing, drying and finishing laundry	1.86E+09	Kg	Market for washing, drying and finishing laundry | washing, drying and finishing laundry | Cutoff, U - GLO
**Bar soap outputs**			
Waste paperboard	979865.8468	Kg	Market for waste paperboard | waste paperboard | Cutoff, U - GB
Wastewater, unpolluted, from residence	1.22E+12	L	Market for wastewater, unpolluted, from residence | wastewater, unpolluted, from residence | Cutoff, U - row
**Hand gel 1 inputs**			
Ethanol, without water, in 99.7% solution state, from fermentation	9.62E+08	Kg	Market for ethanol, without water, in 99.7% solution state, from fermentation | ethanol, without water, in 99.7% solution state, from fermentation | Cutoff, U - GLO
Glycerine	2.68E+07	Kg	Market for glycerine | glycerine | Cutoff, U - RER
Hydrogen peroxide, without water, in 50% solution state	3651569.712	Kg	Market for hydrogen peroxide, without water, in 50% solution state | hydrogen peroxide, without water, in 50% solution state | Cutoff, U - RER

Water, deionised	2.18E+08	Kg	Market for water, deionised | water, deionised | Cutoff, U - Europe without Switzerland
Electricity, medium voltage	1463851.872	Kwh	Market for electricity, medium voltage | electricity, medium voltage | Cutoff, U - GB
Electricity, medium voltage	1829814.84	Kwh	Market for electricity, medium voltage | electricity, medium voltage | Cutoff, U - GB
Electricity, medium voltage	426956.796	Kwh	Electricity voltage transformation from high to medium voltage | electricity, medium voltage | Cutoff, U - GB
Injection moulding	6.29E+07	Kg	Injection moulding | injection moulding | Cutoff, U - RER
Polyethylene terephthalate, granulate, bottle grade, recycled	5.71E+07	Kg	Market for polyethylene terephthalate, granulate, bottle grade, recycled | polyethylene terephthalate, granulate, bottle grade, recycled | Cutoff, U - row
Polyethylene, high density, granulate	5855407.488	Kg	Market for polyethylene, high density, granulate | polyethylene, high density, granulate | Cutoff, U - GLO
Electricity, medium voltage	406625.52	Kwh	Market for electricity, medium voltage | electricity, medium voltage | Cutoff, U - GB
Printed paper, offset	182981.484	Kg	Market for printed paper, offset | printed paper, offset | Cutoff, U - GLO
Transport, freight, lorry 3.5-7.5 metric ton, EURO3	3.19E+08	Kg*km	Market for transport, freight, lorry 3.5-7.5 metric ton, EURO3 | transport, freight, lorry 3.5-7.5 metric ton, EURO3 | Cutoff, U - RER
Transport, freight, lorry >32 metric ton, EURO4	7.65E+11	Kg*km	Market for transport, freight, lorry >32 metric ton, EURO4 | transport, freight, lorry >32 metric ton, EURO4 | Cutoff, U - RER
Transport, passenger car	2.81E+07	Km	Market for transport, passenger car | transport, passenger car | Cutoff, U - RER
**Hand gel 1 outputs**			
Ethanol	9.43E+08	Kg	N/A: elementary flow
Ethanol	1.92E+07	Kg	N/A: elementary flow
Hydrogen peroxide	3578540	Kg	N/A: elementary flow
Hydrogen peroxide	73031.42857	Kg	N/A: elementary flow
Waste paperboard	182981.484	Kg	Market for waste paperboard | waste paperboard | Cutoff, U - GB
Waste polyethylene	5855407.488	Kg	Market for waste polyethylene | waste polyethylene | Cutoff, U - GB
Waste polyethylene terephthalate	5.71E+07	Kg	Market for waste polyethylene terephthalate | waste polyethylene terephthalate | Cutoff, U - GB

Wastewater, from residence	2.45E+08	L	Market for wastewater, from residence | wastewater, from residence | Cutoff, U - row
**Hand gel 2 inputs**			
Glycerine	2.68E+07	Kg	Market for glycerine | glycerine | Cutoff, U - RER
Hydrogen peroxide, without water, in 50% solution state	3651569.712	Kg	Market for hydrogen peroxide, without water, in 50% solution state | hydrogen peroxide, without water, in 50% solution state | Cutoff, U - RER
Isopropanol	8.65E+08	Kg	Market for isopropanol | isopropanol | Cutoff, U - RER
Water, deionised	3.38E+08	Kg	Market for water, deionised | water, deionised | Cutoff, U - Europe without Switzerland
Electricity, medium voltage	1463851.872	Kwh	Market for electricity, medium voltage | electricity, medium voltage | Cutoff, U - GB
Electricity, medium voltage	1829814.84	Kwh	Market for electricity, medium voltage | electricity, medium voltage | Cutoff, U - GB
Electricity, medium voltage	426956.796	Kwh	Market for electricity, medium voltage | electricity, medium voltage | Cutoff, U - GB
Injection moulding	6.29E+07	Kg	Injection moulding | injection moulding | Cutoff, U - RER
Polyethylene terephthalate, granulate, bottle grade, recycled	5.71E+07	Kg	Market for polyethylene terephthalate, granulate, bottle grade, recycled | polyethylene terephthalate, granulate, bottle grade, recycled | Cutoff, U - row
Polyethylene, high density, granulate	5855407.488	Kg	Polyethylene, high density, granulate, recycled to generic market for high density PE granulate | polyethylene, high density, granulate | Cutoff, U - Europe without Switzerland
Electricity, medium voltage	406625.52	Kwh	Market for electricity, medium voltage | electricity, medium voltage | Cutoff, U - GB
Printed paper, offset	182981.484	Kg	Market for printed paper, offset | printed paper, offset | Cutoff, U - GLO
Transport, freight, lorry 3.5-7.5 metric ton, EURO3	3.24E+08	Kg*km	Market for transport, freight, lorry 3.5-7.5 metric ton, EURO3 | transport, freight, lorry 3.5-7.5 metric ton, EURO3 | Cutoff, U - RER
Transport, freight, lorry >32 metric ton, EURO4	7.78E+11	Kg*km	Market for transport, freight, lorry >32 metric ton, EURO4 | transport, freight, lorry >32 metric ton, EURO4 | Cutoff, U - RER
Transport, passenger car	2.81E+07	Km	Market for transport, passenger car | transport, passenger car | Cutoff, U - RER
**Hand gel 2 input**			

2-Propanol (Elementary flows/Emission to air/high population density)	8.47E+08	Kg	N/A: elementary flow
2-Propanol (Elementary flows/Emission to water/unspecified)	1.73E+07	Kg	N/A: elementary flow
Hydrogen peroxide (Elementary flows/Emission to air/unspecified)	3580000	Kg	N/A: elementary flow
Hydrogen peroxide (Elementary flows/Emission to water/unspecified)	73000	Kg	N/A: elementary flow
Waste paperboard	182981.484	Kg	Market for waste paperboard | waste paperboard | Cutoff, U - GB
Waste polyethylene	5855407.488	Kg	Market for waste polyethylene | waste polyethylene | Cutoff, U - GB
Waste polyethylene terephthalate	5.71E+07	Kg	Market for waste polyethylene terephthalate | waste polyethylene terephthalate | Cutoff, U - GB
Wastewater, from residence	3.65E+08	L	Market for wastewater, from residence | wastewater, from residence | Cutoff, U - row
**Liquid soap inputs**			
Soap	4.94E+08	Kg	Market for soap | soap | Cutoff, U - GLO
Electricity, medium voltage	686180.565	Kwh	Market for electricity, medium voltage | electricity, medium voltage | Cutoff, U - GB
Electricity, medium voltage	160108.7985	Kwh	Market for electricity, medium voltage | electricity, medium voltage | Cutoff, U - GB
Injection moulding	2.36E+07	Kg	Injection moulding | injection moulding | Cutoff, U - RER
Polyethylene terephthalate, granulate, bottle grade, recycled	2.14E+07	Kg	Market for polyethylene terephthalate, granulate, bottle grade, recycled | polyethylene terephthalate, granulate, bottle grade, recycled | Cutoff, U - row
Polyethylene, high density, granulate	2195777.808	Kg	Polyethylene, high density, granulate, recycled to generic market for high density PE granulate | polyethylene, high density, granulate | Cutoff, U - Europe without Switzerland
Electricity, medium voltage	152484.57	Kwh	Market for electricity, medium voltage | electricity, medium voltage | Cutoff, U - GB
Printed paper, offset	68618.0565	Kg	Market for printed paper, offset/printed paper, offset/Cutoff, U-GLO

Tap water	1.22E+12	Kg	Market for tap water | tap water | Cutoff, U - Europe without Switzerland
Washing, drying and finishing laundry	1.86E+09	Kg	Washing, drying and finishing laundry | washing, drying and finishing laundry | Cutoff, U - GLO
Transport, freight, lorry 3.5-7.5 metric ton, EURO3	1.29E+08	Kg*km	Market for transport, freight, lorry 3.5-7.5 metric ton, EURO3 | transport, freight, lorry 3.5-7.5 metric ton, EURO3 | Cutoff, U - RER
Transport, freight, lorry >32 metric ton, EURO4	3.11E+11	Kg*km	Market for transport, freight, lorry >32 metric ton, EURO4 | transport, freight, lorry >32 metric ton, EURO4 | Cutoff, U - RER
Transport, passenger car	1.05E+07	Km	Market for transport, passenger car | transport, passenger car | Cutoff, U - RER
**Liquid soap outputs**			
Wastewater, unpolluted, from residence	1.22E+12	L	Market for wastewater, unpolluted, from residence | wastewater, unpolluted, from residence | Cutoff, U - row
Waste paperboard	68618.0565	Kg	Market for waste paperboard | waste paperboard | Cutoff, U - GB
Waste polyethylene	2195777.808	Kg	Market for waste polyethylene | waste polyethylene | Cutoff, U - GB
Waste polyethylene terephthalate	2.14E+07	Kg	Market for waste polyethylene terephthalate | waste polyethylene terephthalate | Cutoff, U - GB
